# Gasdermin D (GSDMD) Is Upregulated in Psoriatic Skin—A New Potential Link in the Pathogenesis of Psoriasis

**DOI:** 10.3390/ijms241713047

**Published:** 2023-08-22

**Authors:** Julia Nowowiejska, Anna Baran, Justyna Magdalena Hermanowicz, Anna Pryczynicz, Beata Sieklucka, Dariusz Pawlak, Iwona Flisiak

**Affiliations:** 1 Department of Dermatology and Venereology, Medical University of Bialystok, Zurawia 14 St., 15-540 Bialystok, Poland; anna.baran@umb.edu.pl (A.B.); iwona.flisiak@umb.edu.pl (I.F.); 2 Department of Pharmacodynamics, Medical University of Bialystok, Mickiewicza 2C St., 15-089 Bialystok, Poland; justyna.hermanowicz@umb.edu.pl (J.M.H.); beata.sieklucka@umb.edu.pl (B.S.); dariusz.pawlak@umb.edu.pl (D.P.); 3 Department of General Pathomorphology, Medical University of Bialystok, 13 Waszyngtona St., 15-269 Bialystok, Poland; anna.pryczynicz@umb.edu.pl

**Keywords:** pyroptosis, GSDMD, gasdermin D, inflammasome, inflammation, cell proliferation

## Abstract

Psoriasis is an important issue in daily dermatological practice. Not only is it an aesthetic defect but it is also a matter of decreased life quality and economic burden. However frequent, the pathogenesis of psoriasis remains uncertain despite numerous investigations. Gasdermins are a family of six proteins. Gasdermin D (GSDMD) is the best-studied from this group and is involved in the processes of inflammation, proliferation, and death of cells, especially pyroptosis. GSDMD has never been studied in psoriatic sera or urine before. Our study involved 60 patients with psoriasis and 30 volunteers without dermatoses as controls. Serum and urinary GSDMD concentrations were examined by ELISA. The tissue expression of GSDMD was assessed by immunohistochemistry. The serum-GSDMD concentration was insignificantly higher in the patients than controls. There were no differences in the urinary-GSDMD concentrations between the patients and controls. Strong tissue expression of GSDMD was significantly more prevalent in psoriatic plaque than in the non-lesional skin and healthy skin of the controls. There was no correlation between the serum-GSDMD concentrations and the psoriasis severity in PASI, age, or disease duration. Taking into consideration the documented role of gasdermins in cell proliferation and death, the increased expression of GSDMD in psoriatic skin may demonstrate the potential involvement of this protein in psoriasis pathogenesis. Neither serum, nor urinary GSDMD can be currently considered a psoriasis biomarker; however, future studies may change this perspective.

## 1. Introduction

One of the greatest mysteries of contemporary dermatology remains psoriasis. However frequent and well-studied, there is still much to discover about its pathogenesis, its potential biomarkers, and the successful methods of its treatment. There are 125 million people suffering from psoriasis worldwide [1], which translates into an affected quality of life and economic burden [2].

What is known about psoriasis pathogenesis is that there are, in general, three links influencing each other mutually: genetic background, immune disturbances, and modifying external stimuli [3,4]. After exposure to environmental stimuli, damage-related molecular patterns (DAMPs) and pathogen-associated molecular patterns (PAMPs) appearing in genetically predisposed individuals stimulate keratinocytes to release antimicrobial peptides (AMPs; e.g., LL-37 and β-defensins), which further activates dendritic cells (DCs). They, on the other hand, secrete different cytokines, which activate several pathways crucial in psoriasis pathogenesis. IL-23 promotes the differentiation of naïve T cells into the Th17 and Th22 subpopulation, whereas IL-12 promotes the Th1 line. Th17 lymphocytes are able to secrete IL-17, which is responsible for the chemotaxis of various cells and the proliferation of keratinocytes. Th22 cells secrete IL-22, which stimulates epidermal proliferation, as well. Th1 cells secrete TNFα and IFNγ [5]. Moreover, DCs also release TNFα and IFNα [6]. IFNγ has the ability to inhibit the apoptosis of keratinocytes [5,7]. It is also necessary to mention the role of inflammasomes. The activation and gathering of the inflammasome complex are induced by the DAMPs or PAMPs via specific pattern-recognition receptors (PRRs). Their assembly can lead to the activation of effector enzymes—caspases—and the release of pro-inflammatory cytokines [8]. It has been reported that PAMPs and DAMPs can activate inflammasomes, which are further documented to be engaged in chronic inflammation and autoimmune diseases [9]. One of such conditions is psoriasis, in which the role of the inflammasomes is well-established [8]. An essential role is also played by IL-1. IL-1β is released due to inflammasome activation [8] and is known to promote the proliferation of T cells and increase IL-17 secretion [5]. Neutrophils must be highlighted, as well, considering they are gathered in the epidermis, forming micro-abscesses and pustules. They may release AMPs during the process called neutrophil extracellular-traps formation (NETosis), in which web-like structures (NETs) are formed. They have also been detected in psoriatic patients [10]. To summarize, psoriasis is characterized mainly by immune disturbances, which lead to the decreased apoptosis of keratinocytes, resulting in hyperproliferation and improper differentiation, as well as chronically sustained inflammatory condition [4]. Considering the significant involvement of many cytokines in psoriasis pathogenesis, there has been a biological therapy treatment developed that targets particular molecules and leads to the inhibition of several immunological pathways. The first group of drugs is TNFα inhibitors (such as adalimumab or etanercept); second—IL-17 inhibitors (such as ixekizumab or secukinumab); and last—IL-23 inhibitors (such as risankizumab and guselkumab) [11,12,13]. They all show great efficacy and significant resolution of psoriatic skin lesions [14]. However, due to such complex cytokine interrelationships, the successful treatment of psoriasis can paradoxically trigger other disorders, such as vitiligo [15,16] or inflammatory bowel disease [17].

Gasdermin D (GSDMD) is a member of the gasdermin-proteins family [18]. GSDMD, similar to other members of this group, has a specific chemical structure: a GSDM N-terminal domain, a linker region, and a GSDM C-terminal domain [18]. This structure allows for their auto-inhibition or activation, which leads to the formation of pores in cellular membranes [19]. It has been shown that all members of the gasdermin family can be cleaved by caspases or granzymes, leading to the release of the N-terminal fragment, which forms the pores in membranes that lead to their death, known as pyroptosis [19]. Different types of caspases may trigger such processes due to various factors [19,20]. Worthy to note, the aim of pyroptosis may be different, which depends on the type of cell, the abundance of gasdermin expression, etc. [19].

GSDMD is probably the best-studied of all the gasdermins. Its encoding gene is located on chromosome 8q24 [21]. GSDMD expression has been noted in many different human tissues, including immune-system cells, the gastrointestinal tract, and the cardiovascular system [19]. GSDMD is engaged in various processes. First, its role in cell death is well-documented. GSDMD is probably most famous for its role in the previously mentioned pyroptosis, which is an inflammatory type of cell death [20]. It is primarily engaged in the non-canonical pyroptosis pathway but, indirectly, it can also be activated by the canonical pathway. Due to several external stimuli, caspases 4 and 5 become active and cleave GSDMD to release the N-terminus, which, in turn, is responsible for pore formation [20]. It is also possible to activate GSDMD through the canonical pathway, where inflammasomes activate caspase 1, which induces the non-canonical pathway and transforms pro-IL-1β into IL-1β, as well [20]. Apart from pyroptosis, GSDMD has also been proven to take part in NETosis [19,22]. Moreover, GSDMD is involved in cell differentiation, coagulation, and thrombosis [19].

Taking into account the documented role of GSDMD in cell death, proliferation, and differentiation [19], we became interested in the investigation of this protein in the context of psoriasis pathogenesis and potential clinical application in this dermatosis.

**Figure 1:**
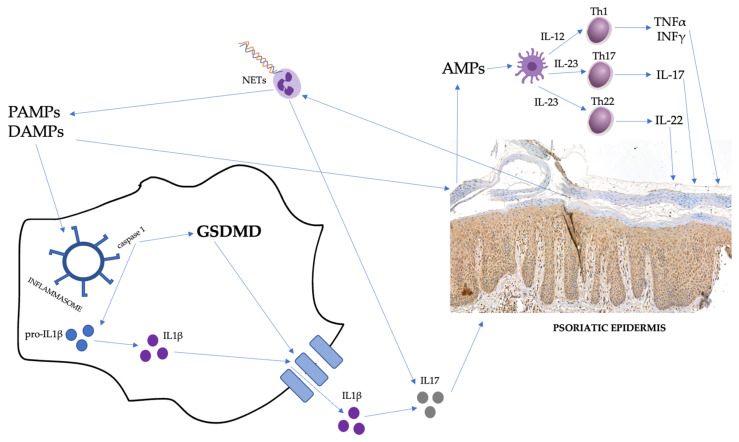
The suspected involvement of GSDMD in psoriasis pathogenesis. PAMPs, pathogen-associated molecular patterns; DAMPs, damage-related molecular patterns; AMPs, antimicrobial peptides; and NETs, neutrophil extracellular traps.

The suspected involvement of GSDMD in psoriasis pathogenesis is presented in Figure 1.

Gasdermins have been poorly investigated in psoriasis so far and both the serum and urinary concentrations and tissue expression of GSDMD in immunohistochemistry have never been studied in this disease. Our team has already assessed all gasdermins in the mentioned biological materials. Apart from the tissue and serum, we chose urine for the investigation because it is easy, painless, and cheap to collect; thus, it would be an ideal fluid for biomarkers’ assessment. Hereby, we present our results regarding the possible role of GSDMD in psoriasis pathogenesis, with reference to its clinical application.

## 2. Results

### 2.1. Serum and Urine

The basic characteristics of the patients and controls are presented in Table 1.

There was no statistically significant difference between the patients and controls in terms of age, gender, or BMI (*p* > 0.05).

**Figure 2:**
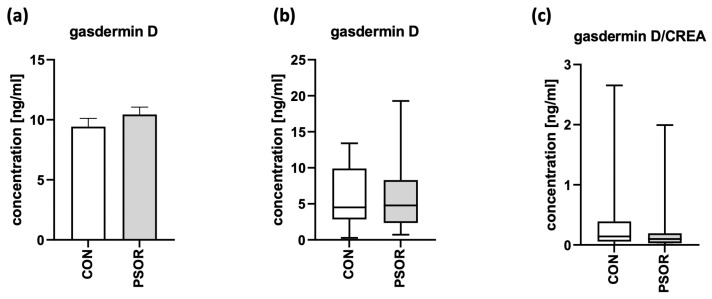
Serum-GSDMD concentration (**a**), absolute urinary-GSDMD concentration (**b**), and urinary-GSDMD/creatinine-concentration ratio (**c**) in 60 patients and 30 controls (ng/mL).

There were no statistically significant differences between the psoriatic patients and controls in terms of the serum and absolute urinary concentrations of GSDMD or urinary GSDMD/creatinine ratio (*p* > 0.05; Figure 2a–c).

**Figure 3:**
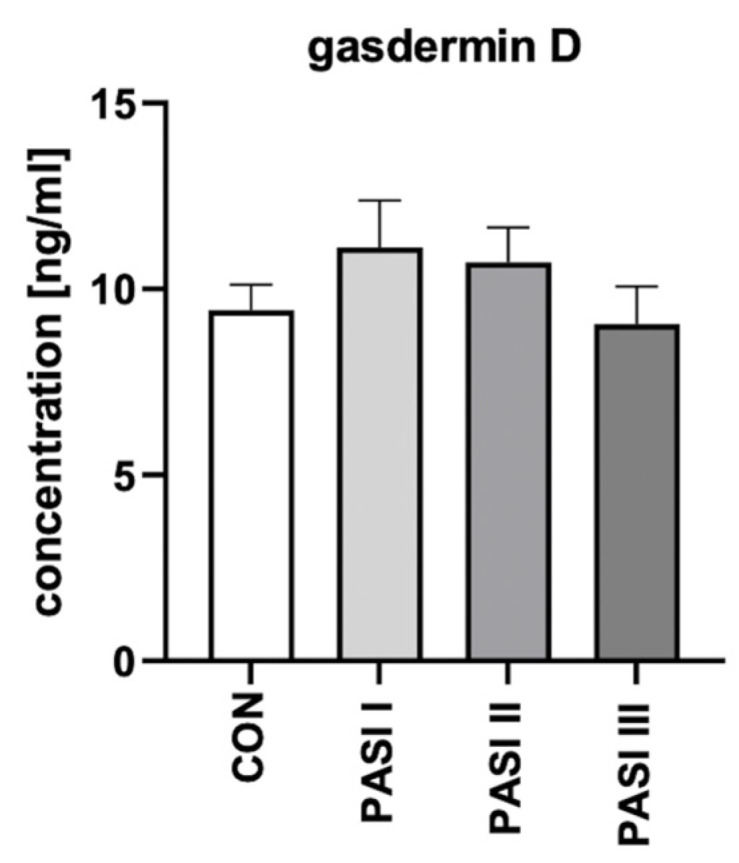
The division of serum-GSDMD concentrations (ng/mL) in 60 patients depending on PASI compared to 30 controls.

After the division of patients into three groups depending on the psoriasis severity in PASI, GSDMD serum concentrations were not statistically significantly different between each group (*p* > 0.05; Figure 3).

There was no significant difference between men and women in serum-GSDMD concentrations (*p* > 0.05). There was no significant correlation between the serum concentration of GSDMD and PASI, BMI, age of patients, or psoriasis duration.

**Figure 4:**
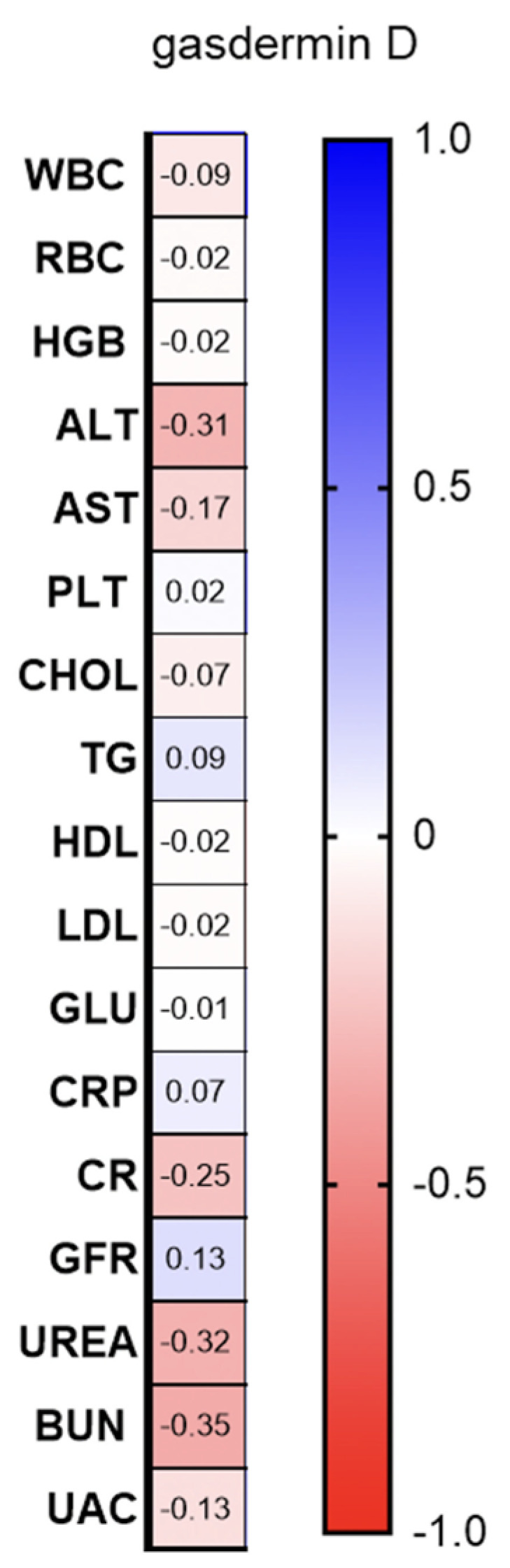
Correlations between serum-GSDMD concentration and basic laboratory parameters. WBC, white blood cells; RBC, red blood cells; HGB, hemoglobin; ALT, alanine transaminase; AST, asparagine transaminase; PLT, platelets; Chol, total cholesterol; TGs, triglycerides; HDL, high-density lipoprotein; LDL, low-density lipoprotein; GLU, fasting glucose; CRP, C-reactive protein; CR, creatinine; GFR, glomerular filtration rate; BUN, blood urea nitrogen; and UAC, uric acid.

There was a negative correlation between serum-GSDMD concentration and ALT activity (R = −0.37; *p* = 0.017; Figure 4).

### 2.2. Tissue

Tissue analysis was performed in 33 patients and 20 controls. Table 2 presents the basic characteristics of this group.

**Figure 5:**
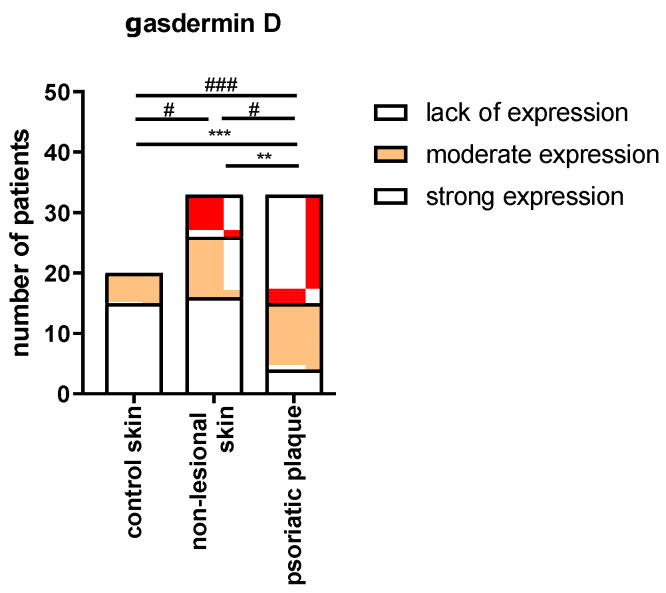
The extent of expression of GSDMD depending on the tissue sample: psoriatic plaque, non-lesional patients’ skin, and healthy skin of controls. **/*** vs. low expression with *p* < 0.01/*p* < 0.001 and #/### vs. strong expression with *p* < 0.05/0.001, respectively.

In the immunohistochemistry, we presented the results as strong, moderate, or lack of the expression of GSDMD in the epidermis. In the group of samples with strong GSDMD expression, the significant majority originated from the psoriatic plaques. The number of samples with strong GSDMD expression from psoriatic plaques was significantly higher compared to the number of samples with strong expression from non-lesional skin in patients (*p* < 0.05) and from the healthy skin in the control group (*p* < 0.001); none of the samples from the control group exhibited strong GSDMD expression. There was no significant difference between the psoriatic plaque, non-lesional skin in patients, and the healthy skin of the controls in terms of the number of samples with moderate GSDMD expression (*p* > 0.05). In the group of samples originating from the psoriatic plaques, there was a significantly lower number of samples that lacked GSDMD expression than in the group from the non-lesional patients’ skin (*p* < 0.01) and from the healthy skin of controls (*p* < 0.001) (Figure 5).

**Figure 6:**
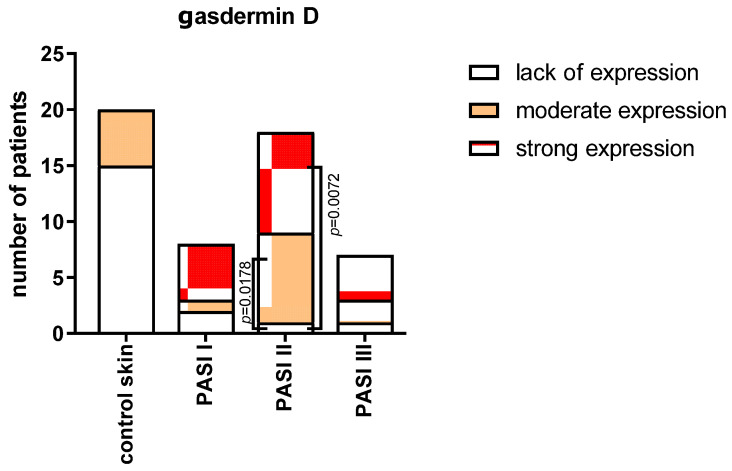
The extent of expression of GSDMD in psoriatic plaque depending on the psoriasis severity in PASI and also compared to controls. The number of samples in each group: PASI I—8, PASI II—18, PASI III—7, and controls—20.

After the division into three subgroups according to PASI (Figure 6), we did not observe significant differences between the extent of GSDMD expression in the samples in each of the PASI subgroups. In the PASI II subgroup, the strong and moderate expressions were significantly more prevalent than the samples lacking this expression (*p* < 0.01 and *p* < 0.05, respectively).

## 3. Discussion

GSDMD has gained much attention lately. Its role has been proven crucial in the pathogenesis of many disorders, such as infections [23], cardiovascular abnormalities [24,25], or acute kidney injury (AKI) [26]. At the same time, its clinical utility as a marker of various disorders has been noted. For instance, GSDMD has been suggested as a marker of bladder cancer [27] and non-alcoholic steatohepatitis (NASH) [28].

Serum and urine, as well as the tissue expression of GSDMD in immunohistochemistry, have never been evaluated in psoriatic patients; thus, we are the first to report on this matter.

The expression of the GSDMD gene has been assessed before in several dermatoses, including psoriasis, on a very small number of samples in the study by Zhang et al. [9]. They revealed that the highest expression was observed in the stratum basale of the epidermis [9]. Furthermore, in another study, it has been reported that the expression of the relative mRNA levels of caspase 4, 5, pro–IL-1β, and GSDMD are all significantly elevated in human psoriatic lesions [29].

However, we did not obtain a statistically significantly higher level of GSDMD in patients’ sera compared to the controls and no significant differences were found. In the urine, we found a higher expression of this protein in psoriatic plaques, which points to its potential role in psoriasis.

Given the proven role of gasdermins in cell proliferation and death, and the initial part of its activation, which is convergent with the initial stage of psoriasis pathogenesis, the upregulation of GSDMD in psoriatic patients compared to the controls without psoriasis demonstrates the potential involvement of this protein in the pathogenesis of psoriasis. It is likely that GSDMD may be its new link. Our suggestion is that the activation of inflammasomes, and then caspases, results in the cleavage of GSDMD, leading to the release of pro-inflammatory cytokines, which are further able to stimulate epidermal proliferation. It has been proven that inflammasome complexes may initiate non-canonical pathway, leading to the release of GSDMD. At the same time, caspase 1 activates IL-1β, which stimulates IL-17, which further promotes epidermal proliferation [5]. To support this theory, there have already been reports of increased inflammasome components in the psoriatic serum, as well as an elevated expression of caspase 1 and IL-1β in psoriatic skin [8], which may further suggest an increased concentration of GSDMD, as well. Another piece of evidence pointing to the rationale of this assumption is the positive influence of particular drugs on psoriasis. The beneficial use of TNFα inhibitors and dimethyl fumarate is documented: First, GSDMD-induced pyroptosis can be inhibited using dimethyl fumarate [19]. Second, there are reports of anti-TNFα-inhibiting NLRP3 inflammasome expression [30].

Another link between psoriasis and GSDMD is NETosis. GSDMD has been documented to participate in this process [22] and it has also been discovered that NETosis plays a role in psoriasis [31]. The side effect of NETosis is that the NETs can become a source of autoantigens and hence trigger the development of autoimmune disorders. Moreover, neutrophils associated with the traps secrete IL-17 [31]—an important cytokine from the point of view of psoriasis. Apparently, there is evidence that the formation of NETs is increased in serum and skin lesions in psoriatic patients. What is more, it correlates with the severity of the disease [31]. The suggested role of NETs in psoriatic patients is the promotion of LL37-DNA complexes’ secretion, which further can stimulate DCs to secrete IFNα, which stimulates the maturation of myeloid DCs [5,31].

Our study indicates that GSDMD is, indeed, supposedly involved in the complex pathogenesis of psoriasis. However, considering our results, serum or urinary GSDMD cannot become a psoriasis biomarker at this time because both were insignificantly elevated in patients. Moreover, we cannot assume that GSDMD can serve as a marker of psoriasis severity because there were no correlations or significant differences in its serum concentration depending on PASI. Considering that our study is the first of this kind, we have no data on psoriatic patients to compare with.

The serum concentrations of GSDMD were independent of gender and psoriasis duration; therefore, GSDMD seems to be potentially engaged in both sexes equally, but currently, it cannot serve as a marker of the time that patients suffer from this dermatosis.

The only correlation we found was the negative one between GSDMD and ALT activity. It may, perhaps, point to the role of GSDMD in liver pathology in psoriatic patients; nevertheless, it is too early to state that and future studies are required. Psoriasis has been linked to non-alcoholic fatty liver disease (NAFLD) and such patients have a two-fold increased odds of concomitant NALFD, which is, in addition, associated with psoriasis severity [32]. The origin of this relationship is probably complex overlapping metabolic disorders [32]. However, GSDMD and pyroptosis have been also suggested in NAFLD and NASH [33]. Apparently, DAMPs are released, which further activates inflammasomes and leads to the pyroptosis of hepatocytes and liver macrophages [33].

As for the limitations of our research, we enrolled a relatively small number of participants of only one ethnicity. The other aspect is the limited data on the kidney metabolism of GSDMD, which may cause difficulties in the interpretation of the results; however, this is why we presented our results both as absolute values of urinary-GSDMD concentrations, as well as a ratio of the urinary/creatinine concentration. Obviously, further studies on bigger samples are needed to better explore this topic.

## 4. Materials and Methods

In total, 60 patients (21 women and 39 men) with active plaque-type psoriasis were enrolled in the study, at the mean age of 50 ± 2.34 years old, as well as 30 sex- and age-matched volunteers without dermatoses and a negative family history of psoriasis. All participants signed informed consent forms before the initiation of the study. The inclusion criterion was adult patients with plaque psoriasis. The exclusion criteria from the study were as follows: age under 18 years old, pregnancy, types of psoriasis other than plaque, dietary restrictions, intake of oral medications at least 3 months prior to the study, malignant neoplasms, and renal impairment. The collection of all the samples took place between 8 July 2020 and 20 April 2023. The severity of the psoriatic skin lesions was assessed using the Psoriasis Area and Severity Index (PASI) and was always performed by the same dermatologist. The study group was divided into three subgroups depending on the severity of the disease: PASI I (PASI < 10)—mild psoriasis, with 16 patients; PASI II (PASI 10–20)—moderate psoriasis, with 30 patients; and PASI III (PASI > 20)—severe psoriasis, with 14 patients. The body mass index (BMI) was calculated as weight/height2. Basic laboratory tests were performed before the study. The study was approved by the Bioethics Committee of the Medical University of Bialystok (APK.002.303.2022 and APK.002.19.2020) and was conducted in accordance with the principles of the Helsinki Declaration [34].

### 4.1. Serum and Urine

The analysis of the serum and urine was performed in 60 psoriatic patients and 30 sex- and age-matched volunteers without dermatoses. Fasting blood samples were taken using vacuum tubes. They were left to clot for 30 min before centrifugation for 10 min at 2000× *g*. Urine samples were taken as first-morning specimens from a mid-stream and they were centrifugated for 10 min at 2000× *g*. The obtained serum and urine were stored at −80 °C until further analysis. Laboratory parameters were measured using routine techniques. GSDMD concentrations were measured with an enzyme-linked immunosorbent assay (ELISA) provided by EIAab^®^ (Wuhan, China, E15480h), with a minimum detectable dose of 0.312–20 ng/mL. The optical density was read at a wavelength of 450 nm. The concentrations were assessed by interpolation from calibration curves prepared with standard samples provided by the manufacturer. All laboratory tests were performed by the same person in standardized laboratory settings.

### 4.2. Tissue Samples

**Figure 7:**
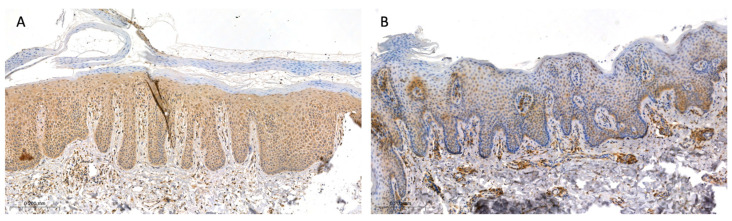
The expression of GSDMD in psoriatic plaque: (**A**) strong, in the whole epidermis, except for the stratum corneum, and (**B**) moderate, in up to 30% of the epidermis (100×).

**Figure 8:**
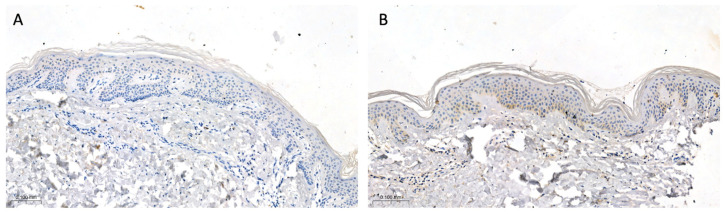
The negative expression of GSDMD (**A**) and the moderate expression of GSDMD (**B**) in the epidermis of the healthy skin of controls (100×).

The tissue sample collection was performed in 33 patients with psoriasis and 20 sex- and age-matched volunteers without dermatoses considering that not every patient agreed to perform the biopsy. This study group was further divided, similar to the subjects who donated serum and urine, into three subgroups depending on the severity of the disease: PASI I (PASI < 10)—mild psoriasis, with 8 patients; PASI II (PASI 10–20)—moderate psoriasis, with 18 patients; and PASI III (PASI > 20)—severe psoriasis, with 7 patients. All participants did not apply any topical drugs on their skin for at least a month before the biopsy. Biopsies were taken from the trunk in all participants with a 4 mm punch after local anesthesia with 2% lignocaine. In the patients, two biopsies were taken: one from the lesional skin—the psoriatic plaque—and the other one from the non-lesional, clinically healthy skin, approximately 2 cm from the psoriatic plaque. As for the controls, one skin sample was taken from the non-lesional skin and the sampling was performed during the surgical removal of benign skin lesions. Tissue samples were then fixed in 10% buffered formalin solution. After preservation, they were embedded in the paraffin blocks and cut into 4 µm sections on silanized slides. The next step was an overnight incubation at 60 °C, deparaffinization, and rehydration of the tissues. Slides were then incubated with 3% hydrogen peroxide solution to block endogenous peroxidase and with protein block to avoid non-specific antibody binding. Next, tissues were incubated with the rabbit polyclonal anti-human GSDMD antibody (dilution 1:50, Sigma-Aldrich^®^, Saint Louis, MI, USA, HPA0444487) for 30 min at room temperature. Then, post-primary block and Novolink polymer were used (Leica Novolink Polymer Detection System^®^, Deer Park, IL, USA). The protein expression was visualized with Novolink DAB solution and cell nuclei with hematoxylin (Leica Novolink Polymer Detection System^®^, USA). The expression of GSDMD was presented using a semi-quantitative method and the reaction was observed in the epidermis as follows: 0—no expression; 1—moderate, up to 30% keratinocytes, except for the stratum basale and corneum; and 2—strong, in the whole epidermis, except for the stratum corneum. Among the psoriatic plaques, 18 patients exhibited strong expression, 12 patients—moderate, and 3 patients—no expression. Among the non-lesional skin samples, 7 patients exhibited strong expression, 10 patients—moderate, and 16 patients—no expression. In the control group, 5 participants exhibited moderate expression and 15 participants—no expression. Figure 7A presents the strong expression of GSDMD, whereas Figure 7B presents the moderate expression of GSDMD in psoriatic plaque (Figure 7A,B). Figure 8 presents the expression of GSDMD in the epidermis of the healthy skin of the controls (Figure 8A,B).

### 4.3. Statistical Analysis

The normally distributed data were analyzed using a one-way analysis of variance (ANOVA) and shown as mean ± SD. The non-Gaussian data are presented as a median (full range) and were analyzed using the non-parametric Kruskal–Wallis test. Student’s *t*-test or the non-parametric Mann–Whitney test was used to compare the differences between the psoriasis group and the control group. The chi-square test of independence was used to test the relationship between the two nominal variables. The correlations between the examined parameters were assessed with Spearman’s rank test. A *p*-value of less than 0.05 was considered to be a statistically significant difference. All analyses were performed in GraphPad 9.4 Prism.

## 5. Conclusions

This is the first comprehensive study on the possible role of GSDMD in psoriatic patients, including its serum and urinary concentrations’ application in the clinical aspect. We proved that GSDMD is significantly most-expressed in psoriatic plaques and insignificantly elevated in the serum of patients with psoriasis compared to controls without psoriasis. Understanding the role of gasdermins in the death and proliferation of cells, we propose their engagement in psoriasis pathogenesis based on the activation of caspases, which results in the cleavage of GSDMD, leading to the release of pro-inflammatory cytokines and epidermal hyperproliferation. Moreover, pyroptosis, in addition to inducing cell death, exacerbates inflammation, which, when chronically sustained, is a feature of psoriatic epidermis. On the other hand, neither serum, nor urinary GSDMD seem, at the moment, to become markers of psoriasis or its severity.

## 6. Patents

Serum GSDMD is the subject of ongoing patent application no P.445837.

## Figures and Tables

**Table 1:** Basic characteristics of patients and controls.

Parameter	Controls (n = 30)	Psoriatic Patients (n = 60)
Sex (M/F)	20/10	39/21 NS
Age (years)	48 ± 2.45	50 ± 2.34 NS
Height (cm)	1.75 (1.5–1.9)	1.71 (1.5–1.9) NS
Weight (kg)	78.40 ± 2.9	85.43 ± 2.5 NS
BMI	25.85 ± 0.77	27.85 ± 0.64 NS

NS, non-significant difference.

**Table 2:** Basic characteristics of patients and controls undergoing tissue examination.

Parameter	Controls (n = 20)	Psoriatic Patients (n = 33)
Sex (M/F)	7/13	21/12 NS
Age (years)	48 ± 3.0	47 ± 3.3 NS
Height (cm)	1.72 ± 0.01	1.72 ± 0.01 NS
Weight (kg)	76.0 ± 3.2	85.28 ± 3.1 NS
BMI ratio	25.31 ± 0.7	27.42 ± 0.7 NS

NS, non-significant.

## Data Availability

Data available upon request from the authors.
